# The role of physical activity and miRNAs in the vascular aging and cardiac health of dialysis patients

**DOI:** 10.14814/phy2.14879

**Published:** 2021-05-27

**Authors:** Miroslava Rabajdova, Ivana Spakova, Aurel Zelko, Jaroslav Rosenberger, Peter Kolarcik, Vladimira Sobolova, Daniel Pella, Maria Marekova, Andrea Madarasova Geckova

**Affiliations:** ^1^ Department of Medical and Clinical Biochemistry Faculty of Medicine Pavol Jozef Safarik University Kosice Slovakia; ^2^ Department of Health Psychology and Research Methodology Faculty of Medicine Pavol Jozef Safarik University Kosice Slovakia; ^3^ Graduate School Kosice Institute for Society and Health Faculty of Medicine Pavol Jozef Safarik University Kosice Slovakia; ^4^ 2nd Department of Internal Medicine Faculty of Medicine Pavol Jozef Safarik University Kosice Slovakia; ^5^ Fresenius Medical Care ‐ Dialysis Services Kosice Kosice Slovakia; ^6^ Olomouc University Social Health Institute Palacky University Olomouc Czech Republic; ^7^ 2nd Department of Cardiology Faculty of Medicine Pavol Jozef Safarik University and East Slovak Institute of Cardiovascular Diseases Kosice Slovakia

**Keywords:** calcification, cardiovascular health, dialysis, exercise, inactivity, microRNA

## Abstract

Cardiovascular comorbidities are independent risk factors for mortality in dialysis patients. MicroRNA signaling has an important role in vascular aging and cardiac health, while physical activity is a primary nonpharmacologic treatment for cardiovascular comorbidities in dialysis patients. To identify the relationships between muscle function, miRNA signaling pathways, the presence of vascular calcifications and the severity of cardiovascular comorbidities, we initially enrolled 90 subjects on hemodialysis therapy and collected complete data from 46 subjects. A group of 26 subjects inactiv group (INC) was monitored during 12 weeks of physical inactivity and another group of 20 patients exercise group (EXC) was followed during 12 weeks of intradialytic, moderate intensity, resistance training intervention applied three times per week. In both groups, we assessed the expression levels of myo‐miRNAs, proteins, and muscle function (MF) before and after the 12‐week period. Data on the presence of vascular calcifications and the severity of cardiac comorbidities were collected from the patients’ EuCliD^®^ records. Using a full structural equitation modelling of the total study sample, we found that the higher the increase in MF was observed in patients, the higher the probability of a decrease in the expression of miR‐206 and TRIM63 and the lower severity of cardiac comorbidities. A reduced structural model in INC patients showed that the higher the decrease in MF, the higher the probability of the presence of calcifications and the higher severity of cardiac comorbidities. In EXC patients, we found that the higher the increase in MF, the lower the probability of higher severity of cardiovascular comorbidities.

## Graphical abstract

miRNA‐206 associated with the myosynthetic signalling pathway that seems to have a positive effect on muscle function and associated with the presence of vascular calcifications and severity of cardiovascular comorbidities among dialysis patients.

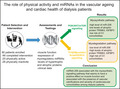

## 1 INTRODUCTION

Cardiovascular disease is the major cause of early morbidity and mortality in patients with end‐stage renal disease on hemodialysis therapy (Chronic Kidney Disease −5^th^ stage on dialysis; CKD‐5D) (Cockwell & Fisher, 2020). More than half of these patients are suffering from several comorbidities with the presence of one or more cardiovascular comorbidities (CVDs). The pathophysiology of cardiovascular and kidney diseases is closely interrelated, and functional impairment in one of these systems is causally related to dysfunctions of the other system, leading to the further failure of both organs (Di Lullo et al., 2015). Hypertension, coronary artery disease, and congestive heart failure directly contribute to elevated cardiovascular mortality in dialysis patients (Cozzolino et al., 2017). Besides these comorbidities, uremic toxicity‐related conditions (albuminuria, anemia, malnutrition, endothelial dysfunction, chronic inflammation, etc.) accelerate the progression of hemodialysis‐induced cardiovascular diseases (Cozzolino et al., 2018); (Subbiah et al., 2016); (Ahmadmehrabi & Tang, 2018). The summary effects of these clinical conditions result in a 20 times higher relative risk of death due to cardiovascular diseases in CKD‐5D patients compared to the general population (Cozzolino et al., 2017).

In the last decade, several microRNA molecules (microRNAs) have been linked to cardiomyocyte development, regeneration and cell homeostasis and considered as a novel source for advances in the diagnosis, prognostics, and therapeutics of cardiovascular and kidney diseases (Brandenburger et al., 2018); (Colpaert & Calore, 2019); (Kölling et al., 2018); (Novak et al., 2014); (Thum et al., 2008). Increased expression of miR‐206 has been found in subjects with arrhythmias (Jin et al., 2019), cardiac hypertrophy (Yang et al., 2015), coronary artery disease, (Westendorp et al., 2012); (Zhou et al., 2015) dilated cardiomyopathy, (Shan et al., 2009) and heart failure(Limana et al., 2011). The concentration of miR‐206 has been negatively correlated with patients’ pulmonary artery systolic pressure and left ventricular end‐diastolic diameter and positively correlated with patients’ left ventricular ejection fraction values (Jin et al., 2017); (Kleeberger et al., 2017). As an independent biomarker, miR‐206 has shown high sensitivity and specificity in the prediction of pulmonary hypertension (Jin et al., 2017). In atherosclerotic plaque samples, miR‐206 was found to suppress the growth and survival of vascular smooth muscle cells (Xing et al., 2017). The overexpression of miR‐206 also inhibited the proliferation of vascular smooth muscle cells and promoted atherosclerosis via the activities of oxidized low‐density lipoprotein in THP‐1 cells (Hu et al., 2019); (Li et al., 2017). Increased expression of miR‐23a has been associated with the presence of arrhythmogenic and hypertrophic cardiomyopathy (Li et al., 2018), cardiac hypertrophy (Lin et al., 2009); (Rooij et al., 2006), pulmonary hypertension (Sarrion et al., 2015), coronary artery disease (Han et al., 2015); (Satoh et al., 2017); (Wang et al., 2016), and aortic calcifications (Babaee et al., 2020); (Toshima et al., 2020). Decreased expression of miR‐23a has been linked with the presence of atrial fibrillation and found in atherosclerotic tissues (Feldman et al., 2017); (Qiao et al., 2020). It is important to mention, that the myoregulative effects of miR‐206 and miR‐23a are affected by interactions with other miRNAs and molecular regulators (like messenger RNAs, short interfering RNAs, etc.) that are able to co‐active or inhibit the metabolic responses (Fu et al., 2020); (Moraes et al., 2017); (Valencia‐Sanchez et al., 2006). The interactions between miR‐1, miR‐133a, and miR‐206 influenced myogenesis, cardiac function, and hypertrophy (McCarthy, 2008); (Novak et al., 2014). A miR‐23a/27a/24‐2 cluster has also been identified as an important mediator of proliferation and differentiation of myoblasts (Mercatelli et al., 2017); (Wang et al., 2012); (Zhao et al., 2020) and protective effects after cardiomyocyte injury (Wu et al., 2020). The evidence has consistently concluded that miR‐206 and miR‐23a play an important role in the pathophysiology of CVDs and vascular calcifications and need to be considered as clinical contributors of increased cardiovascular‐related mortality in CKD‐5D patients.

The biogenetic effects of myo‐miRNAs, such as miR‐206 and miR‐23a, in muscle tissues occur through the activation and deactivation of the PI3 K/AKT/mTOR/FOXO and TGF‐β/Myostatin/Smad signaling pathways. These signaling pathways control and coordinate the cell cycle and proliferation, as well as differentiation processes in myocytes (Kirby et al., 2015); (Novak et al., 2014). The hypertrophic PI3 K/AKT/mTOR signaling pathway could be activated by the binding of insulin‐like growth factor 1 (IGF‐1) on its specific receptor, known as IGF1R (Liu et al., 2018). The cell‐to‐cell transport of IGF‐1 to the receptor is mainly realized by the carrier protein insulin‐like growth factor binding protein 3 (IGFBP3) (Hong & Kim, 2018). The ratio between IGF‐1 and IGFBP3 concentration is referenced as the cellular biological availability of IGF‐1 (Sierra‐Johnson et al., 2009); (Varma Shrivastav et al., 2020). Atrophy processes of myocytes are controlled by the synergic effect of different extracellular signaling pathways, which are activated by transforming growth factor‐β (TGF‐β), myostatin and hepatocyte growth factor (HGF) (Choi et al., 2018); (Wang, 2013). In downstream of the TGF‐β/Myostatin/Smad signaling pathways these factors activate Smad2/3, and Smad2/3 activates the transcription of tripartite motif containing 63 (TRIM63) in the myocyte nucleus (Peris‐Moreno et al., 2020); (Wang & Price, 2020). Tripartite motif containing 63 (TRIM63) has a crucial role in inhibiting the expression of the myogenesis‐responsible genes Pax3/7, MyoD, MyoG, and MyHC and promoting the degradation of myocytes/atrophy processes (Moresi et al., 2010); (Peris‐Moreno et al., 2020). The metabolic effects of miR‐206 and miR‐23a in myocytes are enabled by complex signaling pathways in which IGF‐1, IGFBP3, and TRIM63 are the central mediators of hypertrophic and atrophic cellular signaling.

Intradialytic exercise intervention is considered to be an effective nonpharmacological treatment for cardiovascular comorbidities in CKD‐5D patients (Andrade et al., 2019); (Martin et al., 2018); (Shlipak et al., 2021). For this purpose, exceptional benefits were found for the combination of resistance and aerobic exercises during dialysis. In both types of exercise intervention, the effectiveness of exercise on health‐related risks was strongly dependent on the range of functional and metabolic adaptation of muscle tissue (Celis‐Morales et al., 2017); (Nystoriak & Bhatnagar, 2018); (Sapp et al., 2020). It was found that the adaptability of muscle tissue to exercise intervention should be predicted by the expression of miR‐206 and miR‐23a (Sharma et al., 2014); (Spakova et al., 2020); (Wang et al., 2017).

Resistance training during dialysis is a nonpharmacological stimulus that might counteract disease‐ and treatment‐related decreases in protein synthesis and alterations in the activity of myo‐miRNAs. These beneficial effects might also relate to vascular aging and cardiac health of CKD‐5D patients. However, evidence on the relationships between miR‐206 and miR‐23a expression and downstream signaling, muscle function, the presence of calcifications and the severity of CVDs is limited. To confirm the relationship between cellular signaling, functional adaptation, and patients’ cardiovascular risk factors, we analyzed the associations between changes in muscle function; changes in the expression of miR‐206, miR‐23a, IGF‐1, IGFBP3, and TRIM63; and the presence of calcifications and the severity of CVDs in CKD‐5D patients after the period of physical inactivity and exercise intervention.

## 2 MATERIALS AND METHODS

### 2.1 Participants and intervention conditions

Data for this publication originates from an experimental, two‐group, pre‐post comparative study conducted at three dialysis centers in Slovakia (Fresenius Medical Care Dialysis Services in Kosice, Logman East in Kosice, and Fresenius Medical Care Dialysis Services in Banska Bystrica). The study design and protocol were approved by the Ethics Committee of Pavol Jozef Safarik University in Kosice (approval no. 14 N/2017) and registered at ClinicalTrials.gov (ID:NCT03511924). All methods, assessments and data acquisitions were conducted according to the relevant ethical guidelines and regulations, based on the Declaration of Helsinki (1975, as revised in 2013) and followed the study protocol (Zelko et al., 2019). Informed consent was obtained from all individual participants included in the study. We recruited patients who were diagnosed with end‐stage renal disease (CKD‐5D), older than 30 years of age and who had received maintenance dialysis for at least the last three months. Patients were excluded if they had an acute intercurrent disease, had severe dementia or retardation, lower extremity amputation, or expected mortality exceeding 25% according to the Charlson Comorbidity Index (Charlson et al., 1987). All patients included in the study were treated with online hemodiafiltration using polysulfone dialyzers FX CorDiax600 (Fresenius Medical Care). The dialysis prescription (including duration, dialysate choice, anticoagulation, drug treatment, etc.) was not dependent on participation in the study and was fully under the responsibility of medical director of the dialysis center who adapted it according to the patient´s needs. Filtration volumes and lengths of dialysis therapy were adapted for the patient's treatment according to the clinical results and were comparable between study participants and groups. From the initially included 198 patients, we identified 126 patients as being eligible for participation in the study according to criteria (63.6% eligible patients), 90 of whom agreed to join the study (71.4% response rate). Patients were allocated into the group that underwent intradialytic resistance training (IRT) (EXC, *n* = 57) and the group that remained physically inactive during hemodialysis (INC, *n* = 33). The exercise group (EXC) patients underwent a 12‐week IRT program. All IRT sessions were realized three times per week at dialysis centers, during the course of hemodialysis therapy and were supervised by members of the training team. To enable patients to exercise in a supine position during dialysis, we applied an external source of resistive force generated by elastic bands and plastic balls (TheraBand^®^, Akron, OH, USA). These external loading resources were fixed on the construction of the dialysis bed, and during exercises, patients pulled or pushed against them. IRT sessions were approximately 40 minutes length, composed of 3 minutes warming up, 30 minutes of conditioning and 7 minutes of cooling down and stretching. The program included three exercises: (1) unilateral push and pull of plastic ball, (2) bilateral knee squeeze of plastic ball, and (3) unilateral straight leg raise against the elastic band. To control the patient's training progress during IRT, the numbers of repetitions and series for each exercise were registered on the patient's training log‐book by the training assistant. If the patient´s physical and health conditions allowed, we fulfilled the IRT program in every training session. The progress of the IRT program was patient‐tailored and depended on a patient's physical capabilities. During the first 2 weeks of the IRT, the patient performed an initial program, which consisted of three sets of three exercises (12 up to 15 repetitions of each exercise) of lower extremity muscles. Once a patient was capable to safely complete sessions of the initial program as planned, then the number of repetitions in the next session increased with three repetitions for each exercise. If the patient reached the maximal number (18) of repetitions per exercise during a session, then for the next session the number of sets was increased with one set. If the patient was able to perform five sets with 18 repetitions for each exercise, then we made the IRT harder by applying stiffer elastic band or plastic ball with higher hardness. Vice versa, if the patient failed to complete the entire training session, or had obvious difficulties, the IRT was facilitated by lowering the number in all above steps sequentially. This methodology of training progressivity enabled us to maintain the patient's safety during IRT and ensured the “medium” subjective intensity of training, corresponding to 11–14 out of 20 at the rate of the Borg Scale of Perceived Exertion (Borg, 1982). The dialysis procedure itself may affect a patient´s perception of effort. To maintain patient´s motivation during IRT, training assistants realized extensive counselling included emphasizing the importance of intradialytic exercise and giving positive feedback and commending patients for the training efforts. The inactiv group (INC) patients received standard nephrology care and remained physically inactive during dialysis sessions. Through the 12‐week control period of the intervention, all INC patients maintained their habitual dietary and physical activity patterns.

### 2.2 Muscle function assessments

The maximal isometric forces (1RM) generated by patients during hip flexion (MF) contraction were assessed using a hand‐held dynamometer (Universal digital force gauge HF 500, SAUTER GmbH). During the assessments, patients were in a supine position and held the dominant leg in a straightened position, while the dynamometer was placed proximally to the ankle, on the anterior surface of the lower leg. The patients were instructed to perform a maximal isometric contraction and hold it for 5 seconds. The tests were repeated with 30‐seconds rest intervals, and the higher values of two consecutive tests were used for the analysis as absolute values of 1RM (measure unit: Newton; N). The absolute values of 1RM were subsequently divided by the subject's body weight to determine relative 1RM forces (Newton per kilogram; N/kg). Changes in the relative 1RM forces during MF were calculated for experimental and control conditions as the post‐intervention measure minus the baseline measure (Zelko et al., 2019).

### 2.3 Gene expression analyses/miRNA profiling

Blood samplings for gene expression analyses were carried out in dialysis sessions prior to the start and after the end of the intervention. Venous blood samples were used for collection of blood plasma and were stored at −80°C. During processing in the lab, plasma samples were defrosted on ice, centrifuged for 10 minutes (1000 x g) at 4°C to remove any potential residues. The target miRNAs sample was isolated using the miRNeasy Serum/Plasma Kit (Qiagen). Isolation of the target miRNA was performed according to the valid methodological protocol approved by the kit manufacturer. The concentration and quality control of purity in the isolated miRNA samples was tested using the Qubit microRNA assay kit (Thermo Fisher Scientific).

**TABLE 1:** Sequences of reverse and forward primers used for detection of target miRNA expression levels

Target miRNAs	Mature miRNA Sequence
hsa‐miR206	UGGAAUGUAAGGAAGUGUGUGG
hsa‐miR23a	AUCACAUUGCCAGGGAUUUCC

The samples of total miRNA were processed for specific reverse transcription into cDNA using the TaqMan Reverse Transcription Reagent Kit (Thermo Fisher Scientific) and miR‐specific reverse TaqMan probes (Thermo Fisher Scientific) hsa‐miR‐206 (ID: 000510) and hsa‐miR‐23a (ID: 000399) (Table 1). The cDNA of the specific miRNA target was used in RT‐PCR via the TaqMan Master Mix II no‐UNG kit and complementary forward TaqMan probes (Thermo Fisher Scientific). A Rotor‐Gene Q‐PCR Thermocycler (Qiagen) was used for the analysis of the RT‐PCR expression level. Due to the biological variability of the biological material samples, the analyzed samples were measured in duplicate for each gene of interest. The final miRNA level of miR‐206 and miR‐23a were evaluated by comparative quantification and ΔCT values using the Rotor‐Gene Q Software (Qiagen). The comparative threshold cycle (CT) method, with “housekeeping” references ^ΔCT−40^ and the average ΔCT of the analyzed samples as the endogenous control, was used for quantification of the individual miRNAs (Farina et al., 2014); (Gevaert et al., 2018); (Roberts et al., 2014). After this normalization, the delta threshold cycle (ΔCT) values were used to determine the delta delta threshold cycle (ΔΔCT) and obtain the relative amount of the miRNA to be determined using the formula for relative quantification (target gene 1) = 2^−ΔΔCT(target gene 1)^ (Arocho et al., 2006); (Livak & Schmittgen, 2001).

### 2.4 Protein level detection

The commercially available Human E3 ubiquitin‐protein ligase TRIM63 kits (MyBiosource), Human IGFBP3 ELISA Kit (Abcam), and Human IGF1 ELISA Kit (Abcam) were used to determine the total concentration of TRIM63, IGF1, and IGFBP3 proteins in plasma samples according to the manufacturer's instructions. Subsequently, the concentration of Gal‐3 in each well of the plate was photometrically measured for 5 minutes using a BioTek^TM^ Synergy^TM^ Hybrid Reader H4 plate reader at an absorbance of 450 nm together with subsequent data analysis in the GEN5 program.

### 2.5 Background variables/clinical data

Background variables regarded a) the patient's age and gender, b) body composition parameters (body weight, body height, and body mass index), c) nephrological clinical data containing over‐hydration status, dialysis adequacy (Kt/V) and concentrations of intact parathyroid hormone (iPTH), C‐reactive protein, albumin, phosphates, and calcium. These were extracted from patients’ electronic medical record (EuCliD database). Background variables regarding the prevalence of inactivity were assessed during an investigator‐patient interview. Individual physical activity reports referencing the frequency, duration, and type of physical activities were constructed following the instructions of the Global Physical Activity Questionnaire (Armstrong & Bull, 2006). A patient was considered to be physically inactive if he or she reported less than 3 x 30 minutes of moderate‐intensity physical activity per week (Bull et al., 2020; Thivel et al., 2018).

Clinical data regarded the presence of vascular calcifications and the severity of the CVDs collected from patients’ medical history records during the last 5 years. The severity of CVDs was scored with a “0” if no presence of cardiovascular diseases was recorded, with a “1” if hypertension was recorded or with a “2” if coronary artery disease and/or congestive heart failure were recorded in the patients’ medical records. The presence of vascular calcifications was assessed on the basis of the documented 5‐year history of calcifications in the patients’ medical records. We analyzed the records from routinely performed diagnostic assessments included echocardiography, coronaroangiography, coronary computed tomography angiography, peripheral arteries angiography, abdominal plain X‐ray scan, pelvis X‐ray scan. Every patient had minimal one history record of such assessments and the majority of the study sample had more than three assessments. A score of “0” was assigned to patients if no presence of the calcifications was recorded or a “1” if calcifications were recorded in the patients’ documentation.

### 2.6 Statistical analysis

All statistical analyses of data were carried out using the statistical software package IBM SPSS 22.0 (IBM Corp, 2013). First, we assessed the presence of differences in the baseline variables and compared those between the two study groups using the Student's *t*‐test for continuous variables and tests for categorical (binary) variables. Second, we realized the imputation of missing data in the database. The percentage of missing values across the variables varied between 2.2% and 23.9%. The missing values in the original data table were restored using multidimensional linear regression using fully conditional specification and 10 data sets, in accordance with recommendations of (Myers, 2000) and (Lee & Carlin, 2010). Third, we assessed the relationships between the patients’ age, change in muscle function and changes in the expression profiles of miR‐206, miR‐23a and target protein levels, the presence of vascular calcifications and the severity of CVDs by Spearman correlation tests for the INC and EXC groups. Fourth, we used structural equation modelling to estimate the direct and indirect effects of the change in muscle function with changes in the protein level/miRNA signaling pathways, the presence of vascular calcifications and the severity of CVDs for the total sample (full structural model) and for the INC and EXC groups separately (reduced structural models). We constructed latent variables for these concepts as follows: the change in muscle function was estimated from the change in miRNA expression, the change in miRNA expression from the change in protein level, and finally the change in protein level from the presence of vascular calcifications and the severity of CVDs. Structural modelling was performed using IBM SPSS AMOS software. (IBM Corp, 2013) To increase the model's accuracy and reliability we applied a bootstrap for a target sample of 2000 subjects in the models. The statistical significance level was set at an α level of 0.05.

## 3 RESULTS

Out of the 90 patients included in the study, 64 patients completed all the assessments of the study outcomes. The CONSORT flowchart with the dropouts’ references for the study group is described in a previous report (Zelko et al., 2019). For the present investigation, we included patients who were identified as physically inactive in pre‐intervention assessments, leading to a final number of 46 patients (INC: 26, EXC: 20). The mean age of patients included in the present investigation (*n* = 46) was 66.2 (standard deviation: 9.4). Among these patients, 54% were male and their BMI was 26.1 (5.5) kg/m^2^. Baseline characteristics and their differences between the INC and EXC patients are presented in Table 2. At baseline, we found significant differences between the groups in body mass index, dialysis adequacy, expression of miR‐206, miR‐23a and IGFBP3, and concentration of albumin and calcium.

**TABLE 2:** Baseline characteristics of patients and their comparisons between the groups

Variable	INC (*n* = 26)	EXC (*n* = 20)	*p value*
Age	67.9 (8.9)	63.9(9.9)	0.161
Gender (male/female)	14/12	11/9	0.938
Body mass index (kg/m^2^)	24.3 (4.6)	28.2 (6.5)	0.029^*^
Hip flexion (N)	96.3 (28.5)	107.6 (53.3)	0.397
miRNA−206 (log10)	17.5 (12.0)	42.7 (33.4)	0.013^*^
miRNA−23a (log10)	2132.8 (3944.0)	6557.2 (5139.0)	0.004^†^
IGF1 (pg/ml)	2.6 x 10^5^ (0.54)	2.3 x 10^5^ (0.48)	0.870
IGFBP3 (pg/ml)	11.1 x 10^5^ (0.26)	12.9 x 10^5^ (0.31)	0.045^*^
TRIM63 (pg/ml)	1194.8 (1093.9)	1743.5 (1669.3)	0.228
Dialysis adequacy (Kt/V)	2.0 (0.3)	1.6 (0.4)	0.001^†^
Length of dialysis therapy (months)	45.9 (46.1)	43.0 (47.2)	0.836
Over‐hydration index (%)	12.2 (6.9)	12.3 (6.2)	0.964
C‐reactive protein (mg/l)	13.3 (14.4)	8.3 (10.7)	0.190
iPTH (pg/ml)	392.4 (460.4)	400.8 (375.3)	0.941
Albumin (g/l)	36.9 (4.4)	39.3 (3.0)	0.042^*^
Phosphates (mml/l)	1.5 (0.5)	1.6 (0.5)	0.396
Calcium (mmol/l)	2.3 (0.1)	2.1 (0.2)	0.001^†^
Calcifications (no/yes)	8/18	7/13	0.762
Cardiovascular comorbidities (severity)	2/7/17	5/7/8	0.149

Note

Data are presented as mean ±standard deviation. The miRNA relative expression data are presented as the mean log10 relative quantity (log10) of the respective miRNA ± standard deviation. The protein level data are presented as pg/ml ± standard deviation. The presence of calcifications is coded as no (not presented) or yes (presented). The severity of cardiovascular comorbidities are coded as 0 (not presented), or 1 (hypertension presented) or 2 (coronary artery disease, congestive heart failure presented). *p*‐values are determined by the unpaired Student's t‐test. Differences between the groups significant at *p* < 0.05 are marked by *. Differences between groups significant at *p* < 0.01 are marked by †.

Abbreviations: EXC, experimental condition; IGF1, insulin‐like growth factor 1; IGFBP3, insulin‐like growth factor binding protein 3; INC, control condition; iPTH, intact parathyroid hormone; TRIM63, E3 ubiquitin‐protein ligase.

During the 12 weeks of the strength training intervention in the EXC patients, the maximal force during isometric MF increased by 15.9 N (SD 36.7; pre: 107.6 N, post: 123.5 N; +14.8%). In the INC patients the 12 weeks of physical inactivity resulted in an increase of the maximal isometric force during MF by 3.0 N (SD 26.9; pre: 96.3 N, post: 99.3 N; +3.1%). The relative force during isometric MF increased in the EXC group by 18.4 N/kg (SD 48.8; pre: 139.1 N/kg, post: 157.5 N/kg; +13.2%) and decreased in the INC by 1.5 N/kg (SD 40.3, pre: 141.4 N/kg; post: 139.8 N/kg; −1.1%) (Sharma et al., 2014). The changes in expressions of miR‐206, miR‐23a, IGF1, IGFBP3, and TRIM63 for INC and EXC are summarized in Table 3.

**TABLE 3:** Change in miRNA and proteins expression for the study groups

Variable	INC (*n* = 20)	EXC (*n* = 26)
miRNA−206 (log10)	+14.9 (25.3)	+19.3 (50.2)
miRNA−23a (log10)	+10420.9 (13189.0)	+900.4 (11293.5)
IGF1 (pg/µl)	−0.1 (0.6)	−0.2 (0.6)
IGFBP3 (pg/µl)	−89.0 (486.3)	−5527.8 (20446.9)
TRIM63 (pg/µl)	+24.2 (45.9)	−68.0 (1658.1)

Note

Data are presented as mean change ± standard deviation.

The correlation matrices of the examined variables are presented for the INC (Table S1) and EXC (Table S2) groups separately. For the INC group, the strongest negative correlation was found between changes in concentrations of IGF1 and IGFBP3 (r = −0.84, *p* < 0.01), followed by a positive correlation between patients’ age and the severity of CVDs (r = 0.49, *p* < 0.05). Among EXC patients, we found a strong negative correlation between the change in expression of miR‐206 and change in muscle function (r = −0.51, *p* < 0.05) and a strong positive correlation between the change in expression of miR‐206 and change in expression of TRIM63 (r = 0.50, *p* < 0.05). We also found positive correlations between the change in expression of IGF1 and IGFBP3 (r = 0.47, *p* < 0.05) and between the expression of TRIM 63 and the severity of CVDs (r = 0.46, *p* < 0.05). Other correlations between the parameters did not reach statistical significance.

**FIGURE 1:**
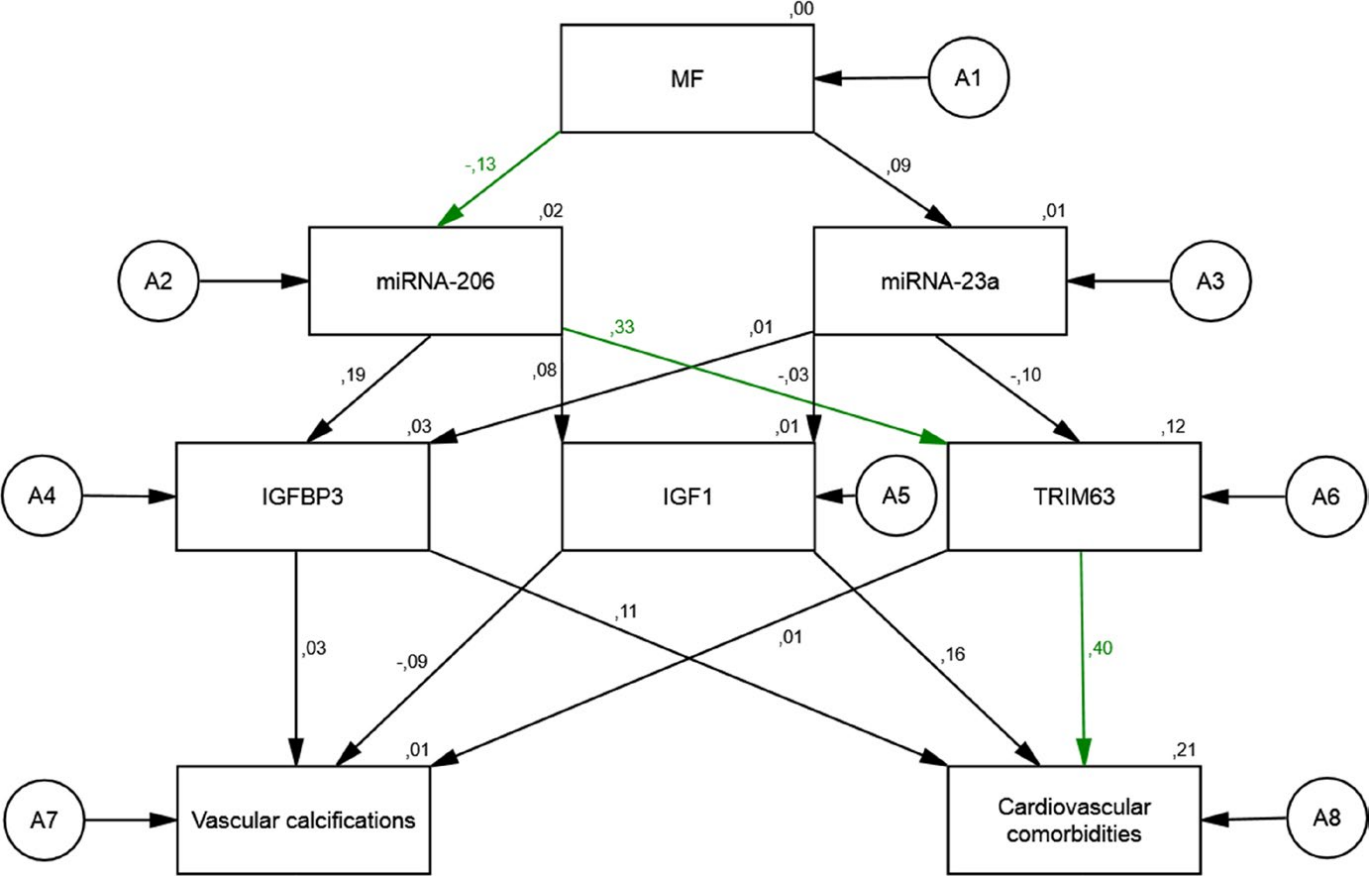
The associations of a change in Hip flexion force on the expression of miRNA‐206, miRNA‐23a, IGF1, IGFBP3, TRIM63 and the presence of vascular calcifications, and the severity of cardiovascular comorbidities in the total sample: a full structural equation model

Figure 1 describes the full structural equation model with one latent variable (MF) and seven constituting variables. The standardized direct effect of change in MF on change in miR‐206 (−0.13) and on change in miR‐23a (0.09) was relatively low. However, the standardized direct effect of change in miR‐206 on TRIM63 (0.33), as well as the standardized direct effect of change in TRIM63 on the presence of cardiovascular comorbidities (0.40), were relatively high. Low values for goodness‐of‐fit indices were obtained (*χ^2^* = 25.44; *df* = 14; *χ^2^*/*df* = 1.82; *p* = 0.030, RMSEA = 0.135). The relations between the latent variables indicate medium cohesion. The resulting regression parameters were low and the model explained a small amount of the variability of the latent variables. The residual variances of endogenous variables were low and regression weights were satisfactory.

In this full structural model representing all patients in the study, change in MF was associated with the observed change in miR‐206. The higher the increase in MF, the higher the probability of a decrease in miR‐206 was observed. The higher the decrease in miR‐206, the higher was the probability of a decrease in TRIM63. The higher the decrease in TRIM63, the lower was the severity of cardiovascular comorbidities observed.

**FIGURE 2:**
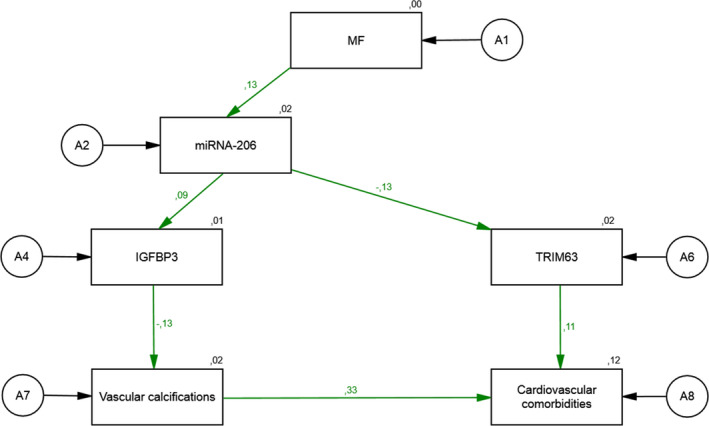
The associations of a change in Hip flexion force on the expression of miRNA‐206, IGFBP3, TRIM63 and the presence of vascular calcifications, and the severity of cardiovascular comorbidities in INC patients: a reduced structural equation model

Figure 2 describes the reduced structural equation model with one latent variable (MF) and five constituting variables containing data from INC patients only. The standardized direct effect of a negative change in MF (a decrease in relative force during isometric MF of about 1.1%) on the change in miR‐206 (0.13) was relatively low. The standardized direct effect of a change in miR‐206 on changes in the protein level of IGFBP3 (0.09) and TRIM63 (−0.13) was relatively low. However, we found strong direct effects of a change in IGFBP3 on the presence of vascular calcifications (−0.13) and the severity of cardiovascular comorbidities (0.33). We also found a standardized direct effect of a change in TRIM63 on the severity of cardiovascular comorbidities (0.11). In this model, high values for goodness‐of‐fit indices were obtained (*χ^2^* = 4.66; *df* = 9; *χ^2^*/*df* = 0.52; *p* = 0.863, RMSEA = 0.000). The relations between the latent variables indicate high cohesion. The resulting regression parameters were high and the model explained an important amount of the variability of the latent variables. The residual variances of endogenous variables were high and standardized regression weights were satisfactory.

In this reduced structural model representing INC patients, a change in MF was associated with the observed change in miR‐206. The higher the decrease in MF, the higher was the probability of a decrease in miR‐206. The higher the decrease in miR‐206, the higher probability of a decrease in the protein level of IGFBP3 and an increase in TRIM63 was observed. The higher the decrease in IGFBP3, the higher the probability of a higher presence of vascular calcifications and a higher severity of cardiovascular comorbidities in INC patients was observed. The higher the increase in TRIM63, the higher was the probability of the presence of severe cardiovascular comorbidities.

**FIGURE 3:**
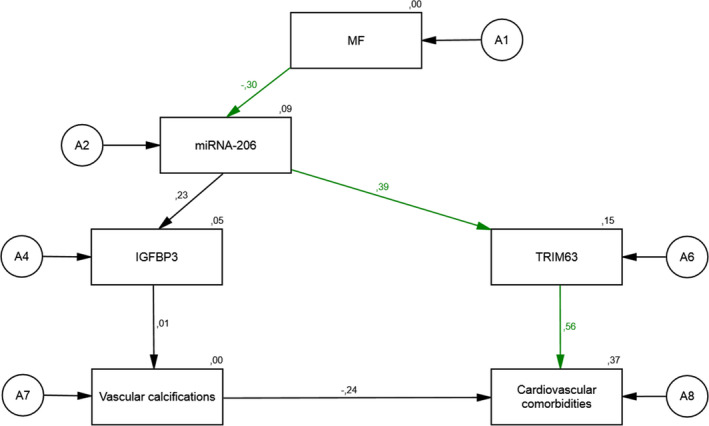
The associations of a change in Hip flexion force on the expression of miR‐206, IGFBP3, TRIM63 and the presence of vascular calcifications, and the severity of cardiovascular comorbidities in EXC patients: a reduced structural equation model

Figure 3 describes the reduced structural equation model with one latent variable (MF) and five constituting variables containing data from EXC patients only. The standardized direct effect of a positive change in MF (an increase in relative force during isometric MF of about 13.2%) on the change in miR‐206 (−0.30) was relatively high. The standardized direct effect of a change in miR‐206 on the change in the protein level of TRIM63 (0.39) was also relatively high. We found strong direct effects of a change in TRIM63 on the severity of cardiovascular comorbidities (0.56). In this model, low values for goodness‐of‐fit indices were obtained (*χ^2^* = 8.73; *df* = 9; *χ^2^*/*df* = 0.97; *p* = 0.463, RMSEA = 0.000). The relations between the latent variables indicate sufficient cohesion. The resulting regression parameters were relatively high and the model explained a high amount of the variability of the latent variables. The residual variances of endogenous variables were high and regression weights were satisfactory.

In this reduced structural model representing EXC patients, a change in MF was associated with the observed change in miR‐206. The higher the increase in MF, the higher was the probability of a decrease in miR‐206. The higher the decrease in miR‐206, the higher the probability of a decrease in the protein level of TRIM63 was observed. The higher the decrease in TRIM63, the lower severity of cardiovascular comorbidities was observed.

In the full factorial and reduced structural models, we did not find any significant associative role of miR‐23a. The change in expression of miR‐23a achieved only weak correlations with the change in muscle function and with changes in the protein levels of IGF‐1, IGFBP3, and TRIM63 in the INC and EXC groups. Reduced structural models for the INC and EXC groups including miR‐23a showed a low standardized direct effect of change in the expression of miR‐23a on the change in MF, IGFBP3, and TRIM63. These models reached low values for goodness‐of‐fit indices and the relations between the latent variables indicate insufficient cohesion (Supplementary Figures S1 and S2).

## 4 DISCUSSION

After a period of physical inactivity in patients, we found that the higher the decrease in muscle function, the higher the probability of the presence of calcifications and the higher severity of cardiac comorbidities. After a period of intradialytic resistance intervention, we found that the higher the increase in muscle function, the lower the probability of higher severity of cardiovascular comorbidities.

We can assume that maximal isometric contraction forces of skeletal muscle cells might be related to the contraction force of all muscle cells and possibly might related also with functions of vascular smooth muscle cells (VSMCs). In the normal physiologic contractile phenotype of VSMCs, physiological blood flow is secured by reliable contractile force of smooth muscle cells. The presence of vascular calcification decreases the VSMCs’ ability to do their physiological work. Calcification of soft vascular tissues is due to dysregulation of Ca^2+^/Pi homeostasis, inflammatory and stress processes, leading to increased CV comorbidities, and accelerated calcifications (Zununi Vahed et al., 2020).

This process is regulated by signaling pathways of myogenesis affected by levels of miR‐206 and miR‐23a levels. MiR‐206 via direct changes in growth factors expression interferes with the binding of them to the receptor, thereby targeting the effect of the Smad pathway and preventing the transcription of the myodegradative genes of TRIM63, MuRF1, and Antrogin‐1 proteins and also preventing HDCA4, FGF23 activation, Ca^2+^ leaching, and muscle tissue calcification (Boettger et al., 2014; Liu et al., 2015). At the same time, miR‐206 promotes the innervation and angiogenesis of atrophied muscle cells, due to calcification. The data obtained by monitoring signaling myohypertrophic and myoatrophic pathways demonstrate the direct effect of myoregulatory miRNAs and the level of myoregulatory proteins on the presence of vascular calcifications and the severity of CVDs, in CKD‐5D patients.

In the group of patients exposed to 12 weeks of IRT, we determined an increase in MF, a decrease in miR‐206, along with a decrease in IGFBP3 and TRIM63 protein levels, which might be considered as contributors of a lower severity of CVDs. Downregulation of miR‐206 might have led to an increase in myogenesis by binding of IGF1 to IGF1R and activation of the PI3 K/AKT/mTOR myosynthetic pathway. An increase in the IGF1/IGFBP3 ratio in favour of IGF1 by a decrease in IGFBP3 levels might have a proteosynthetic effect, as IGFBP3 is a competitive inhibitor of IGF1. The overall positive effect of a decrease in miR‐206 levels is visible in the massive activation of proteosynthesis, thereby attenuating the negative impact of its low level on mitochondrial metabolism, ROS production and impaired innervation based on the described TFGβ/myostatin/Smad/HDAC4/FGF23 pathway. Our analyses further show that the IGF1/PI3 K/AKT/mTOR signaling pathway is stronger in effect, and thus the level of miR‐206 is likely to be due to this myosynthetic signaling.

In contrast, in the group of patients exposed to 12 weeks of physical inactivity, we determined a lower increase in MF, together with a decrease in miR‐206 and the protein level of IGFBP3 and an increase in the protein level of TRIM63, which might be considered as contributors of higher presence of vascular calcifications and higher severity of cardiovascular comorbidities. Thus, the upregulated TFGβ/Myostatin/Smad/TRIM63 myodegradation pathway was likely to have a stronger effect compared to the potency of the IGF1/PI3 K/AKT/mTOR myosynthetic signaling pathway. When analyzing the effect of changes in miR‐23a levels, we did not detect a significant effect on myosynthetic or myodegradative signaling pathways, nor was the prediction of changes in miR‐23a levels and the presence of vascular calcifications and severity of cardiovascular comorbidities confirmed.

Thus, changes in the relative expression levels of the myoregulatory microRNAs, which affect the levels of the myosynthetic proteins IGF1, IGFBP3, and the myodegradative protein TRIM63, are significant predictors of changes in muscle strength in chronically dialyzed patients. In both groups of CKD‐5D patients, we observed different strengths in the prediction of IGF1, IGFBP3, and TRIM63, which influenced the probability of the presence of vascular calcifications and the severity of cardiovascular comorbidities.

Our study has some important strengths. We conducted our study in clinical settings and applied all interventions during regular dialysis therapy. Contrary to other exercise intervention studies carried out in dialysis care settings, to maintain the patients’ safety and preserve validity and reliability we performed muscle function assessments using hand‐held dynamometry. Our study also has some limitations. First, the allocation of patients was realized on the basis of the dialysis center locations, leading to baseline differences in expression of miR‐206 and miR‐23, which could confound the effects of exercise intervention. However, we analyze relationships between expressions of miRNAs and individual responses to exercise and did not find significant associations. Second, we had no concealment of the group allocation of patients during the study, which may have biased outcome assessments. The absence of blinding is a major disadvantage for “exercise/physical activity” intervention designs and cannot be avoided completely during intratherapeutic training interventions. The third limitation of our study is that the extraction of clinical data regarded the presence of vascular calcifications and severity of CVDs. Both clinical variables were collected from patients’ medical records performed in the last 5 years. The missing data from clinical assessments or diagnostics realized intentionally before and after interventions may have led to underestimating these clinical variables among patients and may have biased the collection of data in regard to the presence of vascular calcifications and severity of CVDs.

## 5 CONCLUSIONS

MicroRNA signaling has an important role in vascular aging and cardiac health, while physical activity is a primary nonpharmacologic treatment for cardiovascular comorbidities in dialysis patients. The effectiveness of exercise intervention is strongly dependent on the range of functional and metabolic adaptation in muscle tissue. We found that relations between changes in muscle functions and expression of miR‐206 and miR‐23a, IGF‐1, IGFBP3, and TRIM63 specifically related to exercise and physical inactivity, and to cardiovascular comorbidities of patients. In exercised patients, the higher the increase in MF, the higher was the probability of the decrease in miR‐206, the decrease in miR‐206 and the decrease in the protein level of TRIM63. The higher the decrease in TRIM63, the lower severity of cardiovascular comorbidities was observed. Among inactive patients, the higher the decrease in MF, the higher was the probability of the decrease in miR‐206, the decrease in the protein level of IGFBP3 and the increase in the protein level of TRIM63. The higher the decrease in IGFBP3, the higher the probability of the higher presence of vascular calcifications and the higher severity of cardiovascular comorbidities in patients was observed. The higher the increase in TRIM63, the higher was the probability of the presence of severe cardiovascular comorbidities on these patients.

## CONFLICT OF INTEREST

The authors have no conflicts of interest to declare.

## AUTHORS CONTRIBUTIONS

A.M.G. and J.R. designed the experiments. M.R., I.S., A.Z., V.S., and P.K. performed, analyzed, and interpreted experiments. M.R., I.S., and A.Z. wrote the manuscript. D.P., M.M., and J.R. revised the manuscript for important intellectual content. All authors approved the final version of the manuscript, agreed to be accountable for all aspects of the work and consented to its submission to physiological reports as a research. All persons designated as authors qualify for authorship, and all those who qualify for authorship are listed.

## DATA SHARING STATEMENT

The data supporting the findings of this study are available in repository Zenodo, at https://doi.org/10.5281/zenodo.4461056, reference number: 4461056.

## Supporting information



Fig S1

Fig S2

Table S1‐S2
