# Supplementary figures and images for: The role of physical activity and miRNAs in the vascular aging and cardiac health of dialysis patients
Source: Physiol Rep. 2021 May 27;9(10):e14879. doi: 10.14814/phy2.14879 (PMC8157788; doi:10.14814/phy2.14879)

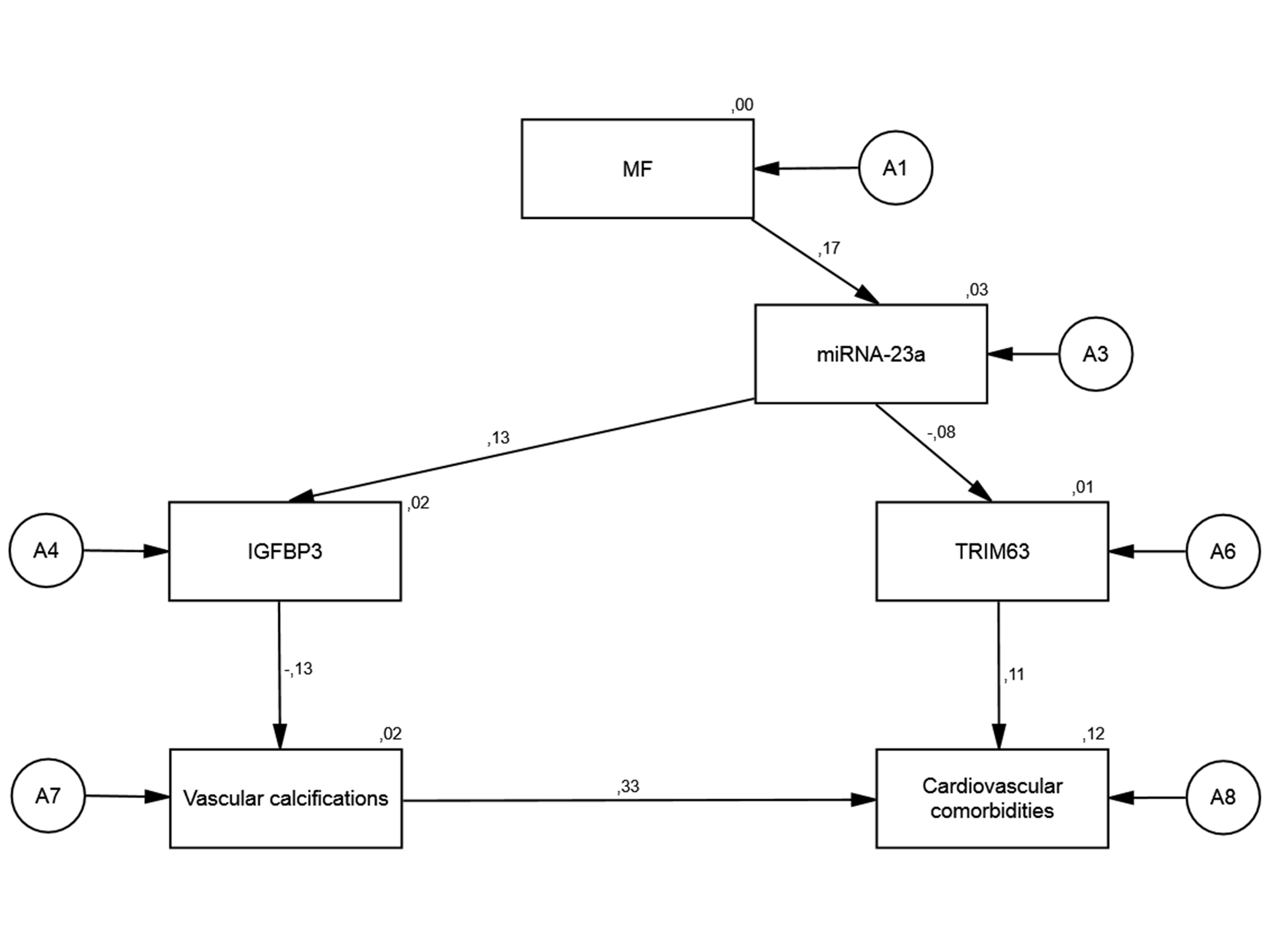

Supplement: Supplementary file 1 — Fig S1 [file PHY2-9-e14879-s002.tif]

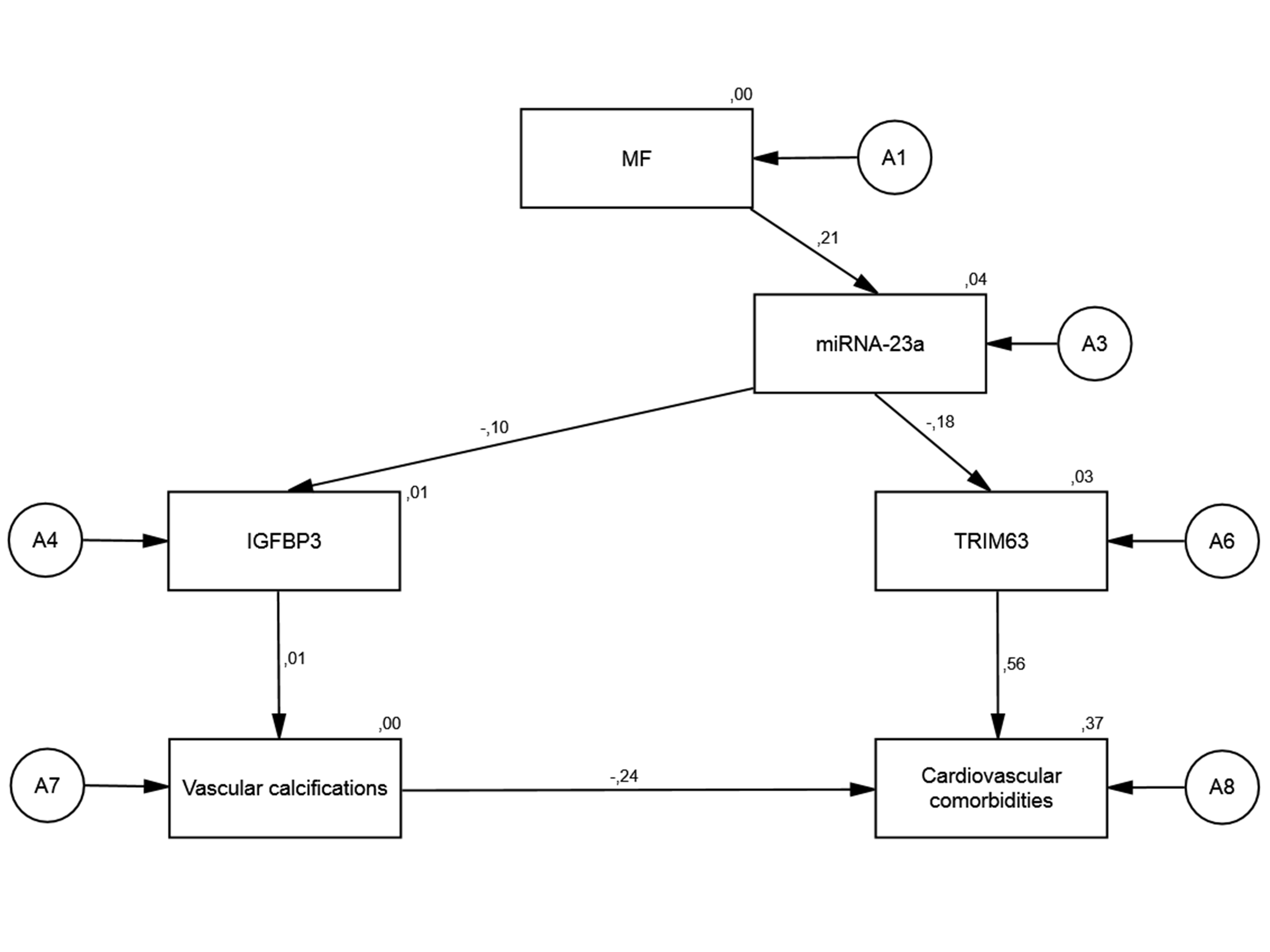

Supplement: Supplementary file 2 — Fig S2 [file PHY2-9-e14879-s001.tif]
