# Supplementary material for: The role of physical activity and miRNAs in the vascular aging and cardiac health of dialysis patients
Source: Physiol Rep. 2021 May 27;9(10):e14879. doi: 10.14814/phy2.14879 (PMC8157788; doi:10.14814/phy2.14879)
Supplement: Supplementary file 3 — Table S1‐S2 [file PHY2-9-e14879-s003.doc]

**Table 4.** Correlation matrix for variables of the INC group

| Parameters | Age | MF | miRNA-206 | miRNA-23a | IGF1 | IGFBP3 | TRIM63 | Calcifications | Cardio-comorbidities |
| --- | --- | --- | --- | --- | --- | --- | --- | --- | --- |
| Age |  |  |  |  |  |  |  |  |  |
|  |  |  |  |  |  |  |  |  |  |
| MF | -0.13 |  |  |  |  |  |  |  |  |
|  |  |  |  |  |  |  |  |  |  |
| miRNA-206 | -0.12 | 0.15 |  |  |  |  |  |  |  |
|  |  |  |  |  |  |  |  |  |  |
| miRNA-23a | -0.10 | 0.15 | 0.22 |  |  |  |  |  |  |
|  |  |  |  |  |  |  |  |  |  |
| IGF1 | 0.19 | -0.37 | 0.13 | 0.09 |  |  |  |  |  |
|  |  |  |  |  |  |  |  |  |  |
| IGFBP3 | -0.06 | 0.35 | 0.00 | -0.03 | **-0.84†** |  |  |  |  |
|  |  |  |  |  |  |  |  |  |  |
| TRIM63 | 0.31 | 0.04 | -0.05 | -0.01 | -0.04 | 0.10 |  |  |  |
|  |  |  |  |  |  |  |  |  |  |
| Calcifications | 0.10 | -0.31 | -0.19 | 0.15 | 0.06 | -0.07 | 0.20 |  |  |
|  |  |  |  |  |  |  |  |  |  |
| Cardio-comorbidities | **0.49*** | -0.07 | -0.19 | -0.10 | 0.18 | 0.06 | 0.16 | 0.28 |  |

Coefficients determined by Spearman correlation tests. Correlations significant at P<0.05 are marked by *. Correlations significant at P<0.01 are marked by ^†^.

**Table 5.** Correlation matrix for variables of the EXC group

| Parameters | Age | MF | miRNA-206 | miRNA-23a | IGF1 | IGFBP3 | TRIM63 | Calcifications | Cardio-comorbidities |
| --- | --- | --- | --- | --- | --- | --- | --- | --- | --- |
| Age |  |  |  |  |  |  |  |  |  |
|  |  |  |  |  |  |  |  |  |  |
| MF | 0.08 |  |  |  |  |  |  |  |  |
|  |  |  |  |  |  |  |  |  |  |
| miRNA-206 | -0.04 | **-0.51*** |  |  |  |  |  |  |  |
|  |  |  |  |  |  |  |  |  |  |
| miRNA-23a | 0.08 | 0.40 | 0.06 |  |  |  |  |  |  |
|  |  |  |  |  |  |  |  |  |  |
| IGF1 | 0.17 | -0.16 | 0.21 | -0.15 |  |  |  |  |  |
|  |  |  |  |  |  |  |  |  |  |
| IGFBP3 | 0.08 | -0.02 | 0.26 | 0.02 | **0.47*** |  |  |  |  |
|  |  |  |  |  |  |  |  |  |  |
| TRIM63 | -0.23 | -0.14 | **0.50*** | -0.01 | 0.28 | -.024 |  |  |  |
|  |  |  |  |  |  |  |  |  |  |
| Calcifications | 0.41 | -0.06 | 0.14 | -0.06 | -0.15 | -0.05 | -0.03 |  |  |
|  |  |  |  |  |  |  |  |  |  |
| Cardio-comorbidities | 0.05 | 0.22 | 0.07 | -0.13 | 0.18 | 0.06 | **0.46*** | -0.26 |  |

Coefficients determined by Spearman correlation tests. Correlations significant at P<0.05 are marked by *.
